# Dodecylphosphocholine Micelles Induce Amyloid Formation of the PrP(110-136) Peptide via an α-Helical Metastable Conformation

**DOI:** 10.1371/journal.pone.0168021

**Published:** 2016-12-08

**Authors:** Simon Sauvé, Yves Aubin

**Affiliations:** 1 Centre for Biologics Evaluation, Biologics and Genetic Therapies Directorate, Health Canada, Ottawa, Ontario, Canada; 2 Department of Chemistry, Carleton University, Ottawa, Ontario, Canada; nanyang technological university, SINGAPORE

## Abstract

A peptide encompassing the conserved hydrophobic region and the first β-strand of the prion protein (PrP(110–136)) shown to interact with the surface of dodecylphosphocholine micelles adopts an α-helical conformation that is localized below the head-group layer. This surface-bound peptide has a half-life of one day, and readily initiates the formation of amyloid fibrils. The presence of the latter was confirmed using birefringence microscopy upon Congo red binding and thioflavin T-binding induced fluorescence. The observation of this metastable α-helical conformer provides a unique snapshot of the early steps of the inter-conversion pathway. These findings together with the body of evidence from the prion literature allowed us to propose a mechanism for the conversion of PrP^C^ to amyloid material.

## Introduction

Transmissible spongiform encephalopathy (TSE) diseases, also known as prion diseases, are associated with the presence of amyloid deposits in brain tissues resulting from the misfolding of the prion protein. The transmissibility of prion diseases is a unique feature of this class of neurodegenerative diseases, and it is mediated by a misfolded intermediate of the prion protein. Prion diseases, including Creutzfeldt-Jakob disease (CJD) and Gerstmann-Sträusler-Scheinker syndrome (GSS) are classified in three categories: sporadic (with no known environmental sources), familial (associated with mutations of the prion protein) and transmitted (from known environmental sources)[1].

The prion protein is a cell surface glycoprotein anchored to the membrane via a glycosylphosphatidylinositol anchor. The mature protein in humans contains 208 amino acids. The N-terminal half of the protein is unstructured in solution and contains eight octa-repeat regions and a highly conserved region (residues 112–128) referred to as the conserved hydrophobic region (CHR). The other half of the polypeptide is a folded globular domain composed of a short β-sheet and three α-helices in which the last two helices are linked via a disulfide bridge. Glycosylation sites are located on helix two and three at residues Asn181 and Asn197.

A detailed mechanism describing the conformational change from the normal cellular form PrP^C^, which is mainly α-helical, to the β-rich conformation, denoted PrP^Sc^, has yet to be developed but elements of the puzzle have been identified. One of them is the involvement of the conserved hydrophobic region in the conversion process. Antibodies targeting PrP(90–120) bind with higher affinity for PrP^C^ compared to PrP^Sc^. This suggests that the solvent exposed CHR in PrP^C^ is participating in the β-structure of the amyloid scaffold[2]. In addition, deletion of this region prevents formation and propagation of PrP^Sc^ [3–5]. Aggregated synthetic peptides of this region are known to cause neurotoxicity[6]. The hydrophobic character of the CHR and the localization of PrP^C^ on the cell surface have directed investigations toward membrane interactions as possible mediators of PrP^Sc^ formation. Mutations that enhance the hydrophobic character of the CHR domain (such as G114V and A117V associated with the GSS syndrome, or the artificial mutations KH-> II [K110I/H111I] and 3AV [A113V/A115V/A118V]) accelerate the onset of neurodegeneration when expressed in transgenic mice[7, 8]. Interactions between the prion protein, the above mutants, and membrane mimetics, such as bicelles[9], and dodecylphosphocholine (DPC) micelles[10], have been shown to produce amyloid fibrils. A detailed structural study of huPrP(110–136) showed the high propensity of this domain to adopt a curved α-helical conformation that is asymmetrically inserted in the detergent micelles[11]. This study suggested that the relative position of the helix within the micelle resulted from a salt bridge between the positively charged guanidine group of the terminal arginine (R136) and the negatively charged phosphate moiety of the detergent headgroup. Finally, a peculiar observation was made. At high peptide-to-micelle ratios, huPrP(110–136) was bound to the micelle surface and it was localized at the hydrophobic-headgroup interface. Here we report that this observation led to the discovery of a key intermediate in the pathway to the conversion of PrP^C^ to amyloid.

## Material and Methods

### Sample preparation and NMR spectroscopy

The preparation of ^13^C, ^15^N huPrP(110–136) NMR sample in DPC micelles and the details of NMR data collection, processing and analysis have been described elsewhere[11]. For this study a total of 28 liters of doubly labelled (^13^C,^15^N) and 25 liters of ^15^N-labelled of bacterial cultures were needed to obtain about 20 mg of purified ^13^C,^15^N-huPrP(110–136) and about 12 mg of ^15^N-huPrP(110–136). Each NMR samples contained 2.8 mg of labelled peptide (2mM) and 2.5 mg of DPC (14 mM) in 0.5 mL of 10 mM NaPi pH 7.5. Data acquisition of correlation experiments using Non-Uniformed Sampling schemes[12–14] was attempted without significant increase in signal intensity or the detection of new correlations. All spectra were referenced relative to DSS and were processed using NMRPipe[15] and analysed with NMRview[16]. Prediction of Φ and Ψ backbone torsion angles and the order parameter (S^2^) from alpha carbon chemical shift was carried out using the web-based version of TALOS+[17, 18] from the National Institute of Health NMR server (http://spin.niddk.nih.gov/bax/nmrserver).

### Preparation and Congo Red staining of amyloid fibrils

The protocol used for Congo red staining was based on Prusiner[19] and Lührs[9]. The aggregated material (white solid) collected from NMR samples containing 1mM of labelled ^13^C-^15^N- or ^15^N-huPrP(110–136) and 5.6 mg/mL (14 mM) of DPC was used for these experiments. The aggregates were resuspended by agitation and then mixed with an equal volume of 50 μM Congo red (Sigma-Aldrich, Saint-Louis, MO) staining solution and 10 mM sodium phosphate pH 7.5. The mixture was incubated with agitation at room temperature for 10 minutes before separation of the solid fraction by centrifugation. The precipitate was red while the supernatant was colourless. The pellet was washed three times with an equal amount of buffer (10 mM sodium phosphate, pH 7.5) to remove non-specific staining. Stained aggregates were transferred to microscopy slides, previously rinsed with ultrapure water. A cover glass was then placed over and sealed on the edges with nail polish to prevent drying of the sample.

### Microscopy

Analysis and photographs were obtained using a Zeiss Axiophot microscope equipped with an Axiocam camera (Zeiss, Thornwood, NY) and two polarizers (Chroma Technology Corp, Bellows Falls, VT). Birefringence was observed by placing the sample between two orthogonally positioned polarizers. Rotating the sample by 45^o^ was carried out to show that the light was refracted in different direction depending of the amyloid fibril orientation. Photographs were taken after calibration of the microscope with black and white reference.

### Binding of Thioflavin T

Aggregated NMR samples and sample components (detergent, peptide and buffer) were tested for thioflavin T (ThT) binding fluorescence, in order to confirm the presence of amyloid-like material[20, 21]. Samples of 2.8 mg/ml (7 mM) and 28 mg/ml (70 mM) of DPC micelles, and 1mg/ml huPrP(110–136) in 10 mM sodium phosphate pH 7.6 were tested. Sufficient amounts of these samples were added to a 50 μM solution of ThT (Sigma-Aldrich, Saint-Louis, MO) to obtain a final concentration of 40 μM, and incubated for 20 min at room temperature. The emission spectrum of ThT was recorded using a SpectraMax i3 Multi-Mode Microplate Detection Platform running SoftMax Pro v6.3 software (Molecular Devices, Sunnyvale, CA). The excitation wavelength used was 440 nm and the emission spectrum was recorded from 465 to 505 nm.

## Results

**Fig 1:**
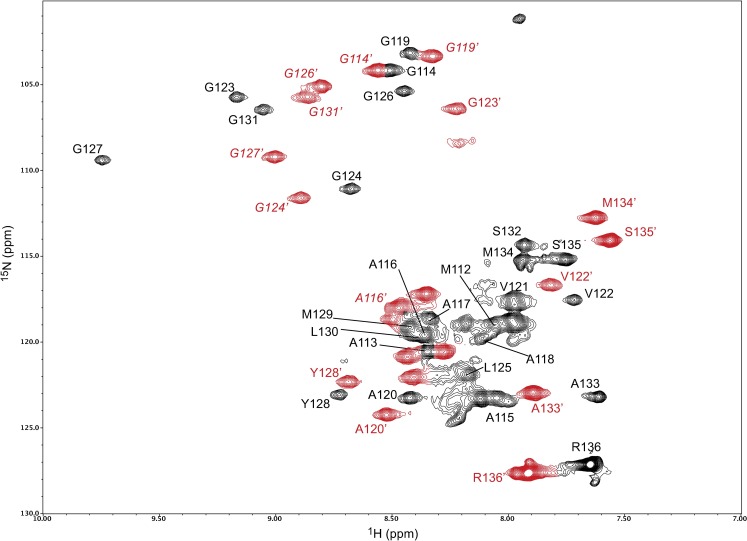
Two-dimensional NMR spectrum of micelle-bound huPrP(110–136). The resonance assigned 2D ^1^H-^15^N HSQC spectrum at 600 MHz of 2 mM ^13^C,^15^N-huPrP(110–136) in 10 mM sodium phosphate pH 7.6 with 14 mM DPC recorded at 37^°^C. The black and red contours correspond to the micelle-inserted and the surface-bound species, respectively.

**Fig 2:**
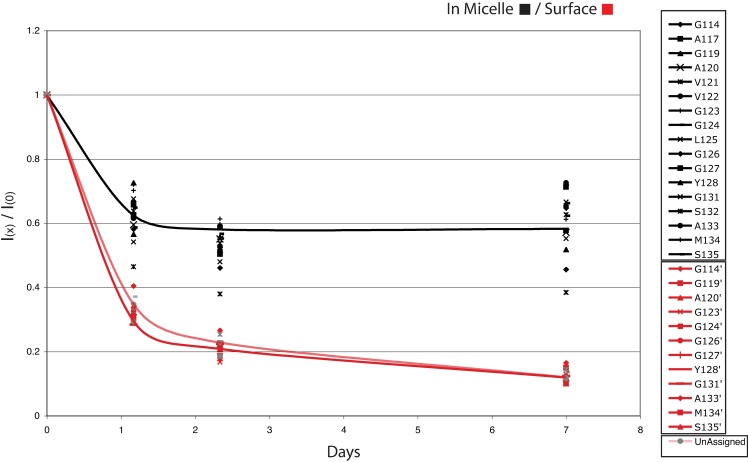
Decrease of the ^1^H-^15^N Signal intensity of the two conformers in the presence of DPC micelles as a function of time. Signal curves that could not be assigned unambiguously where labeled Unassigned.

The initial intent of this study was to characterize the conformation of huPrP(110–136) interacting at the surface of DPC micelles (red signals in Fig 1) observed in our previous report[11]. The spread of resonances for this surface species indicated that the conformation was structured. The NMR data collection was impeded by a significant loss of signal resulting from the precipitation of the peptide. Only a single 3D data set (HNCACB or CBCACONH) could be recorded during the lifetime of a sample. Several attempts were made to collect various 3D-HNCA 3D-HNCOCA in order to obtain a complete set of data for a complete assignment, each time burning 2.8 mg of labeled peptide. Nevertheless, we could obtain unambiguous backbone assignments for 17 out of the 26 residues. Considering that the peptide is very soluble in water, the presence of a white precipitate readily suggested the formation of amyloid-type material. Also, a 2D-HSQC spectrum recorded after recording a three-dimensional experiment (~48h) showed that the surface-bound species had practically disappeared while most of the signal intensities of the inserted peptide seemed less affected. A determination of the half-life of the sample by measuring a time course of signal decay (Fig 2) showed that after only 24h, nearly 80% of the signal intensity of the surface species has disappeared, while the micelle-inserted peptide retained an average of 70% of its signal intensity. Most of the signal loss was observed on the first day.

In an attempt to complete the assignment, we have recorded triple resonance experiments using the non-uniform sampling approach (NUS), with the hope of gaining a higher overall signal-to-noise ratio per unit time and detect unobserved correlations. Two experiments, 3D-HNCO and 3D-CBCACONH, were recorded with the NUS protocol from the manufacturer library sequences with a sparse sampling of 25, 35 and 50% of the total data points in the indirect dimension. After reconstruction and processing, no new inter-residue correlations were observed. Using chemical shifts arguments, and proximity of a given amide pair resonance on the HSQC between the two species; we could deduce four more assignments.

### Conformation and localization of the surface-bound peptide

**Fig 3:**
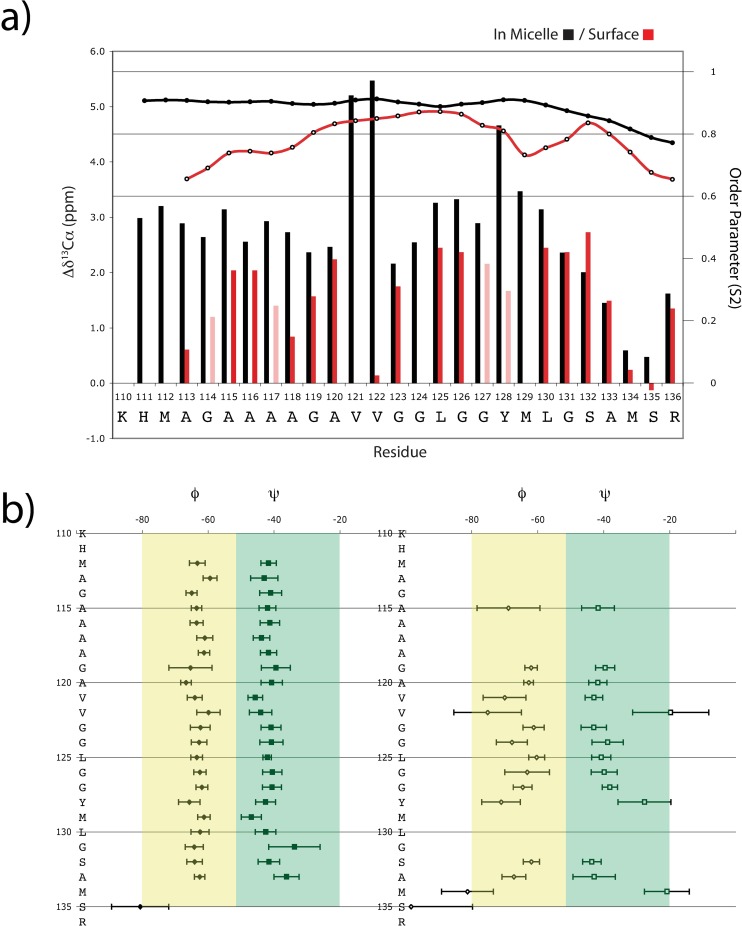
**Analysis of the alpha carbon chemical shifts of the micelle-bound peptide** (A) Carbon alpha secondary chemical shift indicates that the surface population rapidly adopts a flexible alpha-helical conformation prior to aggregating and forming amyloid-like material. Δδ^13^Cα on the left y-axis and the order parameter S^2^ in the right y-axis are displayed as a function of the amino acid sequence for the micelle-inserted population and the surface population in black and red, respectively. Residues that could not be sequentially assigned unambiguously but are positioned in regards to their close proximity in amide chemical shifts are displayed in light red to further show that the alpha helical conformation is observed all over the peptide sequence. (B) The backbone dihedral angle Φ and Ψ (and their standard deviation) generated by TALOS+ are displayed as a function of the amino-acid sequence for the micelle-inserted (left) and the surface population (right).

With a 24 hours sample half-life, a high-resolution structure of the surface-bound peptide could not be determined. A rapid inspection of the secondary chemical shifts suggested that NOE measurements would only waste more labeled peptide without providing geometrically significant distance constraints. Therefore a model was produced that took into accounts the secondary chemical shifts of ^13^Cα (Fig 3A), and a prediction of the backbone torsion angles (Φ,Ψ) and an associated order parameter (S^2^) with the available assignments using TALOS+ (Fig 3B). Overall, the surface-bound peptide displays secondary shifts that are almost as large (average Δδ^13^Cα = 1.5 ppm) as those measured for the corresponding residues in the sequence of the micelle-inserted α–helical peptide (average Δδ^13^Cα = 3.0 ppm) at the C-termini half of the peptide, while residues of the palindromic sequence (113-AGAAAAGA-120) show somewhat smaller values. These clearly suggest that the peptide adopts an α–helical conformation with a looser fold upon binding to the micelle surface. This is also supported by the order parameter (S^2^) predicted by TALOS+. The order parameter predicted for residues of the surface-bound peptide that are less exposed to the paramagnetic agent have an average S^2^ of 0.85, while residues that are more exposed or in the polar layer order parameter falls within 0.65–0.75. In comparison, residues in the hydrophobic core of the micelle-inserted peptide have higher values of S^2^ of 0.88. The significance of this looser fold may simply be that the conformation is made of helical domains with flexible links between them. Predictions of S^2^ values coupled with the near-zero secondary shifts for Val-122 and the lack of a ^13^Cα signal for V-121 suggests a random coil conformation that is dynamic.

**Fig 4:**
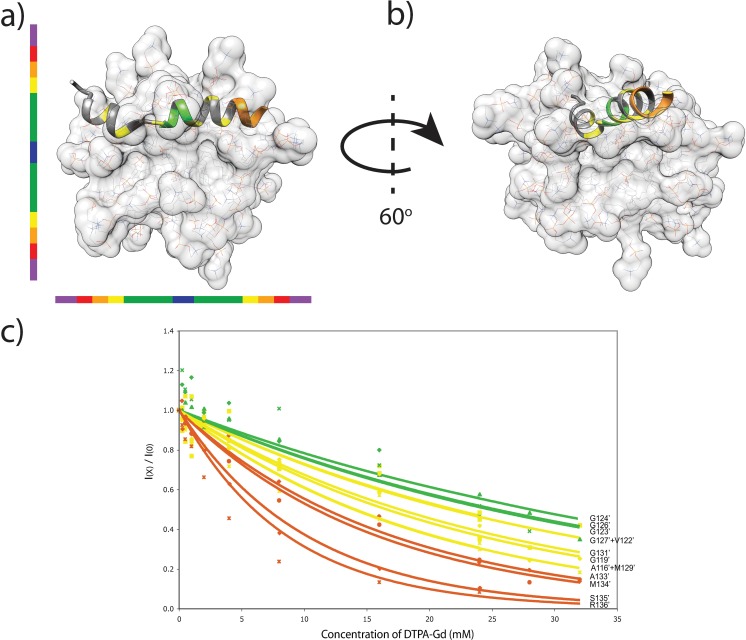
Localisation of the surface-bound peptide to dodecylphosphocholine micelles. The surface-bound population (a and b) lies at the hydrophobic-headgroup interface of the micelle. The depth of the model in the micelle was determined with PRE titration data (c) to manually position both conformers in a DPC micelle by using a color-coded depth gauge, where dark blue represents the centre of the micelle. Note that for clarity, the inserted peptide, which must be present for the surface-bound species to bind, is not displayed in panels ‘a’ and ‘b’.

Unfortunately, the short lifetime of samples prevented a more complete and rigorous characterization of the peptide motions. This observation of transition from random coil in solution to an α–helical conformation at the membrane surface has been well characterized in other amyloidogenic peptide such as α-synuclein[22–24]. To illustrate the NMR data, a model of the surface-bound peptide was generated manually using Chimera[25] by using the Δδ^13^Cα data and the predicted backbone torsion angles (Φ,Ψ) (Fig 4). The peptide was also manually positioned on the micelle based on the PRE data from our previous study[11] using the α–helical micelle-inserted peptide model as a depth gauge.

### Excess of huPrP110-136 is turned over to amyloid in presence of DPC micelles

**Fig 5:**
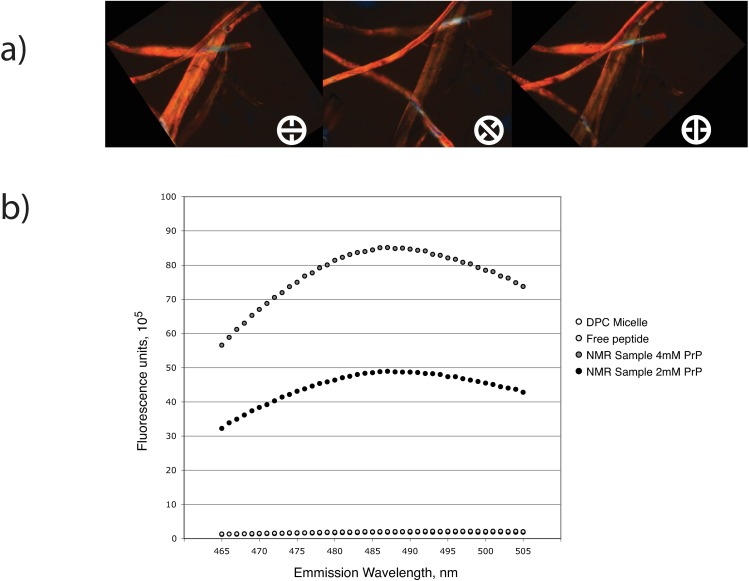
**Surface-bound peptide forms amyloid** (A) Congo red staining and (B) thioflavin T binding induced fluorescence of huPrP(110–136) aggregates. The aggregated material generated in NMR samples shows birefringence under cross-polarized light, indicative of amyloid-like material. Thioflavin T is a dye that becomes fluorescent upon binding to amyloid material.

The white precipitate in NMR samples was tested for the presence of amyloid fibrils. Microscopic observation under cross-polarized light of the collected peptide aggregates after Congo red staining was birefringent (Fig 5A), revealing similar physicochemical properties as reported for the amyloid produced from full length PrP protein interacting with phospholipids bicelles[9] and the amyloid depositions in TSE-infected brains[1].

Birefringence could be observed in different area revealed by the orientation of the crossed polarizers. Three microscopic observations were obtained from three different sample preparations in order to confirm the presence of amyloid material. Considering that optical microscopy observations of birefringence can be difficult to obtain, and that it may not be sufficient to ascertain the presence of amyloid aggregates with this approach[26], we obtained further confirmation with the measurement of the extrinsic fluorescence of thioflavin T (Th-T) upon binding to amyloid fibrils[26, 27]. The emission spectrum (Fig 5B) clearly shows that amyloid fibrils were formed in the presence of DPC micelle under the same sample conditions used for NMR spectroscopy. Also, the average emission over 465–505 nm correlates to the amount of material used in the preparation of the NMR sample. The average intensities of 44X10^5^ RU for the sample containing 2 mM peptide nearly doubled to 77X10^5^ RU for the 4 mM NMR sample, indicating that all the excess peptide (i.e. not inserted into the micelle but bound to the surface) was converted into amyloid material. In contrast, following excitation at 440 nm, no emission was recorded for the free peptide or DPC micelles as a result of a lack of binding of the dye.

## Discussion

The NMR spectrum in Fig 1 shows that, at high peptide-to-detergent ratio, the water-soluble huPrP(110–136) peptide can interact in two different ways with DPC micelles. HuPrP(110–136) inserts into the detergent micelles while adopting a stable α-helical conformation, or it is localized at the headgroup-hydrophobic core interface of an occupied micelle (Fig 4) in a metastable helical conformation prior to the formation of amyloid fibrils.

The micelle-huPrP(110–136) interactions can be explained in the following terms. Backbone amides and carbonyls interactions with the bulk water are easily disrupted in favour of intra-peptide hydrogen bonds during helix formation. This conformation must be stabilised by the micelle environment since all side-chains between residues 111 and 132 are non polar. If the peptide is allowed to fully insert in the micelle, it becomes trapped into a very stable α-helical conformation. In fact, NMR samples made for structural studies of this species were stable for more than a year[11]. In high peptide-to-micelle ratio situation, the peptide readily populates the inside of the micelle with the inserted α-helical conformation, while the excess stays at the headgroup-hydrophobic-core interface. In this environment, hydrophobic and hydrogen bond interactions are available to stabilise this looser conformation. It is noteworthy that Supattapone has reported that various classes of host-encoded cofactor molecules such as phosphaditylethanolamine and RNA molecules are required to form and maintain the specific conformation of infectious prions[28]. Indeed, analysis of the Cα chemical shift, and its associated predicted order parameters, indicates that the conformation is made of two α-helical domains with a more flexible α-helical segment within the palindromic sequence (113-AGAAAAGA-120), and a well-folded helix at the C-terminal (residues 123–136). This conformational behaviour at the micelle surface shares striking similarities with α-synuclein at the surface of SDS-micelles[22, 24]. Upon interaction with detergent micelles, the intrinsically disordered α-synuclein folds into two helices where the lowest values of ^13^Cα secondary shifts are correlated with the flexible residues in the linker between the N- and C-helices segments[24] from a generalised order parameter determined from backbone relaxation measurements. Also, a Gly-Ala-Val motif is present in α-synuclein at the junction of the two helical domains and has been proposed to be required for fibril formation[29]. This motif straddles the end of the N-terminal helix and includes the valine in the helix linker (119-GAV-121). But, in comparison with α-synuclein, the surface-bound species of huPrP(110–136) is transitory. This surface-bound species is a metastable intermediate that has two possible fates. The peptide first inserts into the micelle and then, if the micelle is already populated with an inserted peptide, it converts to β-sheets to form amyloid fibrils with other surface peptide molecules. It is noteworthy that while the inserted peptide is very stable, the process of fibril formation involving the surface-bound conformation can drive the inserted α-helix out of the micelle, converting a part of that population to β-amyloid (Fig 2). The amount of the inserted α-helix decreases until the surface-bound population has been expended. This indicates that the surface helix is a very reactive species that drives the formation of the products, β-amyloid fibrils.

### PrP(110–136) peptide vs full length prion protein

**Fig 6:**
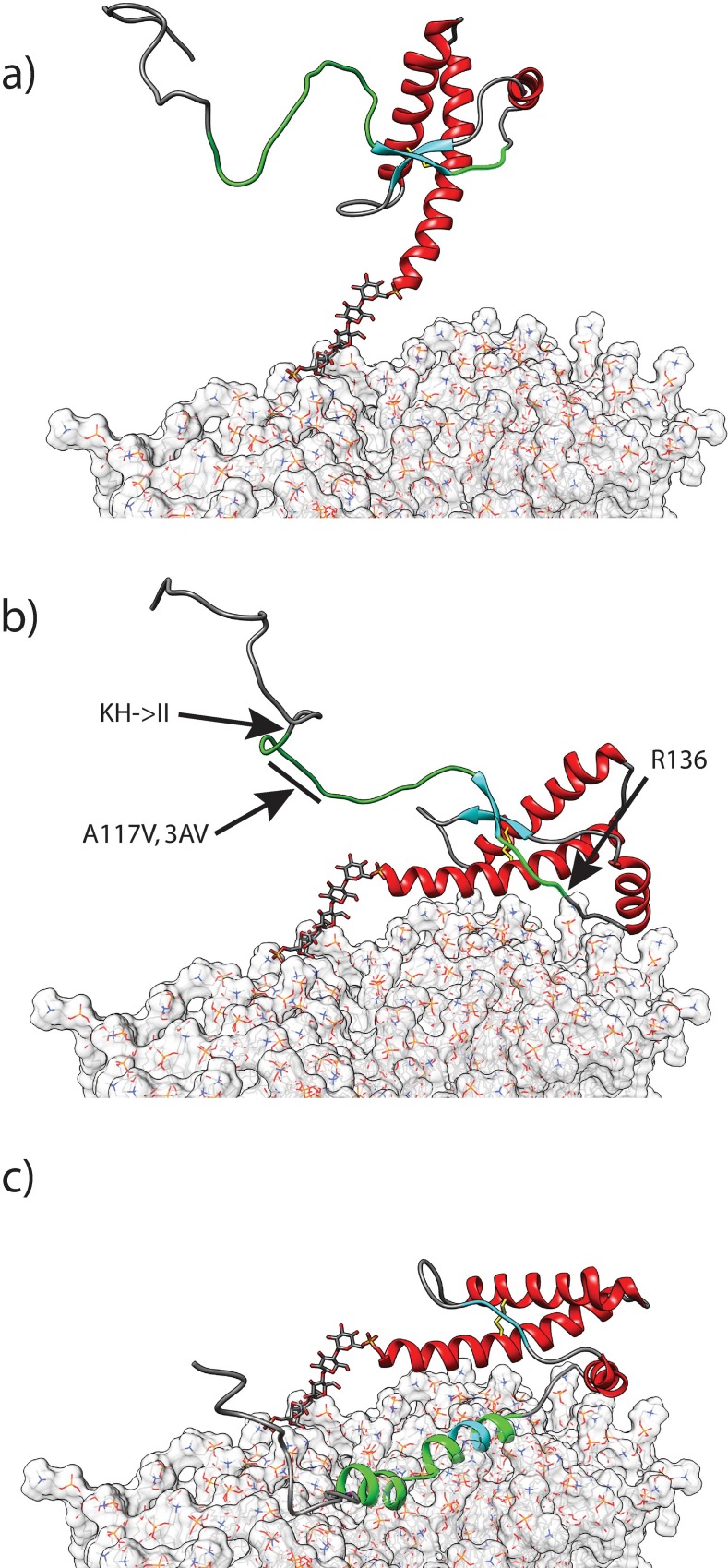
A model of huPrP(90–230) [1QLX.pdb] at the surface of a DMPC bilayer[30] attached with a GPI anchor. (A) The normal huPrP(90–230) at the membrane surface. Residues 110–136 are coloured in magenta. The β-sheet is held by three H-bonds between 129-MLG-131 and 161-VYY-163 that acts as a safety catch (coloured in light blue). (B) Membrane interactions can take place if mutations known to increase hydrophobicity of the CHR are present. These would loosen or break the β-sheet allowing the segment 110–136 to interact with the surface, including the formation of a salt-bridge between R136 and the phospholipid headgroup. (C) Once this metastable conformer is formed, it readily interacts with other such conformers to form amyloid fibrils.

The membrane interactions observed with huPrP(110–136) involve the conserved hydrophobic region and arginine-136 via a probable salt-bridge with the detergent phosphocholine headgroup[11]. The lack of micelle interactions under similar conditions (pH 7.6) with a shorter peptide, huPrP(110–129), supported the requirement for this salt-bridge. The membrane interactions observed with huPrP(110–136) are not favoured with the full-length wild-type prion protein. The small two-strand β-sheet involving residues 129-ML-130 in the peptide acts as a safety catch to hinder two interactions: the formation of the salt-bridge (R136), and the membrane interaction of the CHR. Factors that can loosen or release the safety catch, thus lowering this barrier, could allow membrane interaction leading to a reactive intermediate that would readily convert and participate in the formation of amyloid fibrils. A series of the GPI-anchored prion protein models was generated to illustrate the proposed mechanism of interaction with the membrane surface that is based on our observations (Fig 6).

This mechanism provides an explanation for the observed pathological effects of mutations (KH-> II [K110I/H111I] and 3AV [A113V/A115V/A118V])[7]. The proposed concept of the safety catch is further supported by NMR studies of DPC micelles interactions with wild type mouse-PrP(90–231), and mutants[10]. The wild type showed no chemical shifts changes (Δδ^13^Cα) in the presence of DPC micelles while the KH->II and 3AV mutants interacted strongly leading to precipitation of the samples, thus preventing complete characterisation of the interactions. Mutation A117V, associated with the Gerstmann-Straüssler-Scheinker syndrome, produced a number of chemical shift changes (Δδ^13^Cα) upon addition of DPC micelles.

## Conclusion

The interaction between DPC micelles and huPrP(110–136) resulted in the insertion of the peptide into the detergent micelle with an α-helical conformation. Once the core of the micelle is occupied, excess peptide could then interact with the micelle surface resulting in the formation of a metastable intermediate. The half-life of this species was sufficiently long to model the peptide-micelle interaction, thus giving a snap-shot at the onset of the conversion pathway of huPrP(110–136) to amyloid. These observations indicates that the normal membrane-anchored prion protein can undergo a drastic conformational change leading to the formation of amyloid plaques provided that appropriate conditions are encountered. While the peptide is unhindered, we are proposing that the full-length protein is held back by a simple safety catch that prevents the interactions observed here. Therefore, conditions suitable for the formation of a metastable intermediate can be the increased hydrophobicity through mutations in the palindromic region, low pH and electrostatic interactions, both have been observed in *in vitro* studies[9, 28]. Here, we have opened a door that has been kept shut, therefore shedding light in the mechanism of conversion. We trust that the observation of the metastable conformer can be a starting point to molecular dynamic studies that will venture farther into this unknown conversion pathway.
